# Differential Yellow Fever Susceptibility in New World Nonhuman Primates, Comparison with Humans, and Implications for Surveillance

**DOI:** 10.3201/eid2701.191220

**Published:** 2021-01

**Authors:** Natália C.C. de Azevedo Fernandes, Juliana M. Guerra, Josué Díaz-Delgado, Mariana S. Cunha, Leila delC. Saad, Silvia D. Iglezias, Rodrigo A. Ressio, Cinthya dos Santos Cirqueira, Cristina T. Kanamura, Isis P. Jesus, Adriana Y. Maeda, Fernanda G.S. Vasami, Júlia de Carvalho, Leonardo J.T. de Araújo, Renato Pereira de Souza, Juliana S. Nogueira, Roberta M.F. Spinola, José L. Catão-Dias

**Affiliations:** Instituto Adolfo Lutz, São Paulo, Brazil (N.C.C. de Azevedo Fernandes, J.M. Guerra, J. Díaz-Delgado, M.S. Cunha, S.D. Iglezias, R.A. Ressio, C. dos Santos Cirqueira, C.T. Kanamura, I.P. Jesus, A.Y. Maeda, F.G.S. Vasami, J. de Carvalho, L.T. de Araújo, R. Pereira de Souza, J.S. Nogueira);; Universidade de São Paulo, São Paulo (N.C.C. de Azevedo Fernandes, J.M. Guerra, J. Díaz-Delgado, J.L. Catão-Dias);; Texas A&M Veterinary Medical Diagnostic Laboratory, College Station, Texas, USA (J. Díaz-Delgado);; Centro de Vigilância Epidemiológica Prof. Alexandre Vranjac, São Paulo (L.D.C. Saad, R.M.F. Spinola)

**Keywords:** yellow fever, yellow fever virus, viruses, arboviruses, liver, differential susceptibility, new world nonhuman primates, humans, surveillance, immunohistochemical analysis, PCR, outbreak, zoonoses, Brazil

## Abstract

A major outbreak of yellow fever (YF) occurred in Brazil during 2016–2018. Epizootics in New World nonhuman primates are sentinel events for YF virus circulation. However, genus-specific susceptibilities and suitability for YF surveillance remain poorly understood. We obtained and compared epidemiologic, histopathologic, immunohistochemical, and molecular results from 93 human and 1,752 primate cases submitted during the recent YF outbreak in Brazil (2017), with the support of the Brazilian National YF Surveillance Program. We detected heterogeneous YF-associated profiles among the various genera of primates we analyzed. *Alouatta* primates were the most reliable sentinel; *Sapajus* and *Callicebus* primates had higher viral loads but lower proportional mortality rates. *Callithrix* primates were the least sensitive, showing lower viral loads, lower proportional mortality rates, and no demonstrable YF virus antigen or extensive lesions in liver, despite detectable viral RNA. These differences in susceptibility, viral load, and mortality rates should be considered in strategic surveillance of epizootics and control measures for YF.

Yellow fever (YF) is a zoonosis caused by YF virus (YFV; family *Flaviviridae*, genus *Flavivirus*) that has 2 established cycles in South America: urban and sylvatic. The sylvatic cycle is maintained by forest canopy mosquitoes (*Sabethes* spp. and *Haemagogus* spp.) and New World primates (NWPs); humans are accidental hosts (*1*,*2*). During 2016–2018, YF reemerged in Brazil, posing new threats with major epidemic waves in areas with low viral circulation, although without evidence of the urban cycle (*3*). The national surveillance program in Brazil for YF primarily relies on YF investigation in deceased free-ranging NWPs (*4*). Surveillance of epizootics in NWPs plays a pivotal role for deployment of prevention actions, emphasizing immediate vaccination of susceptible human populations (*5*).

Brazil has broad and heterogeneous NWP (suborder Platyrrhini) diversity: 5 families, 21 genera, and 176 species (*6*). Nevertheless, knowledge of YF in NWP species is limited; most efforts have focused on serologic testing of the species *Leontopithecus chrysomelas* (*7*), *Alouatta* spp. (*8*,*9*), *Cebus* spp.(*10*), and *Saguinus* spp. and *Saimiri* spp. (*10*). Only howler monkeys (*Alouatta* sp.) have well-documented liver involvement in consequence of natural YF infection (*11*) and are considered reliable sentinels of YFV circulation, because they show higher susceptibility to YF than humans and develop a fatal hepatic failure with massive cellular death (*1*,*4*,*12*). Howler monkeys and laboratory NWP models usually have hepatic changes similar to those found in humans (*3*,*11*): massive necrosis/apoptosis associated with Councilman–Rocha Lima bodies, steatosis, and mild inflammatory infiltrates (*13*,*14*), although diverging pathologic features might also be seen (*3*). Further knowledge of YF pathogenic aspects is needed to guarantee that samples from various species of NWPs would be adequately used for YF surveillance purposes to ensure appropriate diagnoses and subsequent public health responses. In addition, this knowledge will clarify the effect of YF in the wide range of NWPs in Brazil.

During the last YF outbreak in Brazil (2016–2018), we observed differences among genera not only in YF prevalence but also in YF viral load, as reported (*15*). These differences might have implications for NWP as amplifying hosts or as reservoirs in YF cycle. The term reservoir refers to an animal with persistent infection, sometimes without clinical signs, and sufficient amount of pathogen to act as source of infection. Amplifier, although used as a synonym, is a broad term referring to a host with high viral load and source of infection (*16*). Thus, we hypothesized that there are different genus-specific susceptibilities among NWPs that could affect YF surveillance/monitoring. To reduce this knowledge gap, we characterized and compared the histopathologic signature of YF-associated liver disease, viral antigen detection by immunohistochemical analysis (IHC), and molecular findings in samples from humans and NWPs infected by YFV that were received during 2017 at Adolfo Lutz Institute (São Paulo, Brazil).

## Methods

### Data and Sample Collection

We obtained formalin-fixed, paraffin-embedded and fresh frozen (−70°C) liver samples from NWPs and humans from São Paulo state, Brazil, that were submitted for YF diagnosis to the Adolfo Lutz Institute during 2017, according to the Brazilian National Surveillance Program of YF by the Ministry of Health of Brazil (*4*). The Adolfo Lutz Institute is an official laboratory for the diagnosis of YFV in humans and primates. NWP samples came from standardized necropsies performed by local surveillance agents. Epidemiologic (carcass location and date) and biological (genera, sex, age) data were obtained from notification files (Sistema Nacional de Agravos de Notificação) sent with the samples. Cases designated as *Cebus* spp. were reclassified as *Sapajus* spp., according to Alfaro et al. (*17*). Only NWP cases with genera identified and formalin-fixed, paraffin-embedded liver tissue were included in this study. For humans, samples from patients with suspected or confirmed YF who died were obtained; only cases with quantitative reverse transcription PCR (qRT-PCR) results from fresh liver samples were included. Adverse vaccine effects and transplant cases were excluded. All procedures were approved by the Animal Use and Research Ethical Committees of the Adolfo Lutz Institute (CEUA-IAL no. 11/2016 and CEP-IAL no. 3.121.328–caaee 96138818.0.0000.0059) and the Instituto Chico Mendes de Conservação da Biodiversidade protocol 50551–3.

### Spatial Analysis

Cases positive by IHC or qRT-PCR were tabulated and plotted. Plots were made by using a map of São Paulo state and QGis software (https://qgis.org).

### Histopathologic and Immunohistochemical Analyses

Protocols and procedures were conducted in the enhanced laboratory Biosafety Level 2 facility of the Adolfo Lutz Institute. All formalin-fixed, paraffin-embedded liver tissue samples were processed and stained with hematoxylin and eosin for histopathologic examination. IHC was performed according to our laboratory protocols. Liver tissue sections were subjected to antigen retrieval in a pressure cooker in citrate buffer for 3 min (120°C, pH 6.0) and then incubated overnight with polyclonal anti-YF (mouse hyperimmune antiserum against wild strain; Núcleo de Doença de Transmissão Vetorial, Virology Center, Adolfo Lutz Institute) (*3*,*12*). Signal amplification was performed by using the Horseradish Peroxidase–Conjugated Polymer Detection System (REVEAL Biotin-Free Polyvalent; Spring Bioscience Corp., https://www.cmocro.com) and visualized by using diaminobenzidine (D-5637; Sigma-Aldrich, https://www.sigmaaldrich.com) and counterstaining with Harris hematoxylin. In selected instances, amplification was performed by using AP conjugated polymer (MACH4 Universal AP Polymer Kit; Biocare Medical, https://biocare.net) and visualized by using fast red chromogen (WARP RED chromogen kit; Biocare Medical). Known NWPs and human positive and negative control tissues with omitted first-layer antibody were included.

NWP cases were classified according to the distribution, extent, and nature of microscopic findings after staining with hematoxylin and eosin as described (Appendix Table 1). For IHC, cases were classified as positive, negative, or inadequate on the basis of YFV antigen detection and varying degrees of typical YF-associated lesions. Inadequate classification refers to highly autolyzed/decomposed cases with lack of immunolabeling. Human cases were classified as full spectrum of YF-associated hepatic lesions or other histologic patterns. We provide a detailed description of other histologic patterns.

**Table 1:** Yellow fever–related mortality and infections rates for different genera of NWPs, Brazil*

Genus	Mortality rate, no. IHC positive/no. tested (%)	Infection rate, no. IHC or qRT-PCR positive/no. tested (%)	p value
*Alouatta*	437/708 (61.72)	445/708 (62.85)	0.8
*Callicebus*	8/28 (28.57)	9/28 (32.14)	1
*Callithrix*	10/921 (1.09)	25/921 (2.71)	0.015
*Sapajus*	13/95 (13.68)	13/95 (13.68)	1

*IHC, immunohistochemical analysis; NWPs, New Word primates; qRT-PCR, quantitative reverse transcription PCR.

### Molecular Analysis by Using qRT-PCR

Total RNA was extracted from fresh frozen liver by using the QIAamp RNA Blood Mini Kit (QIAGEN, https://www.qiagen.com), according to the manufacturer’s instructions. Amplification of YFV fragments were performed by using a described protocol (*18*) that targets the highly conserved 5′ noncoding region of the genome (112 bp) and is based on a TaqMan qRT-PCR protocol. Quantification cycle (Cq) was used as a reference for viral load (in a standard curve with YFV vaccine 17D, a titer of 10^5^ PFU/mL had a mean Cq = 16, and 1 PFU/mL had a mean Cq = 35.6).

### Statistical Analysis

Data were tabulated and analyzed by using Portal Action (http://www.portalaction.com.br) and R software (https://www.r-project.org). For categorical data, we used χ^2^ or Fisher exact test (2-tailed) tests. For continuous data, after using the Shapiro-Wilk normality test, we applied the Kruskal-Wallis test. Agreement between IHC and qRT-PCR was calculated by using the Cohen kappa contingency coefficient. Sensitivity, specificity, positive predictive value, negative predictive value, and accuracy of histologic analysis were calculated; inadequate cases obtained by IHC were excluded. The IHC detection limit (qRT-PCR Cq value) was obtained by using receiver operating characteristic curve analysis. Correlation of nonparametric data was calculated by using the Spearman test, and a standard curve was created. Statistical difference was defined as p<0.05.

## Results

### NWPs

We analyzed 1,752 NWPs: 413 (23.6%) were female, 638 (36.4%) were male, and 701 (40%) did not have sex identified. Distribution by genus was 921 (52.6%) *Callithrix* spp., 708 (40.4%) *Alouatta* spp., 95 (5.4%) *Sapajus* spp., and 28 (1.6%) *Callicebus* spp. *Alouatta* and *Sapajus* species had higher proportions of males than did *Callithrix* and *Callicebus* spp. (p<0.01). We compiled a population profile, including sex and age distribution by genus (Appendix Table 2), and a spatial distribution of positive NWPs, all from São Paulo state, by IHC, qRT-PCR, or both (Appendix Figure 1).

All cases had IHC results. Of 1,752 cases, 468 (26.7%) were positive for YF: 437 (61.7%) of 708 *Alouatta* spp., 8 (28.6%) of 28 *Callicebus* spp., 13 (13.7%) of 95 *Sapajus* spp., and 10 (1.1%) of 921 *Callithrix* spp. Of the remaining cases, 1,171 (66.8%) were negative and 113 (6.4%) had inadequate results because of autolysis or absence of liver samples. No age (p = 0.55) or sex (p = 0.72) difference between positive and negative groups was detected.

**Figure 1:**
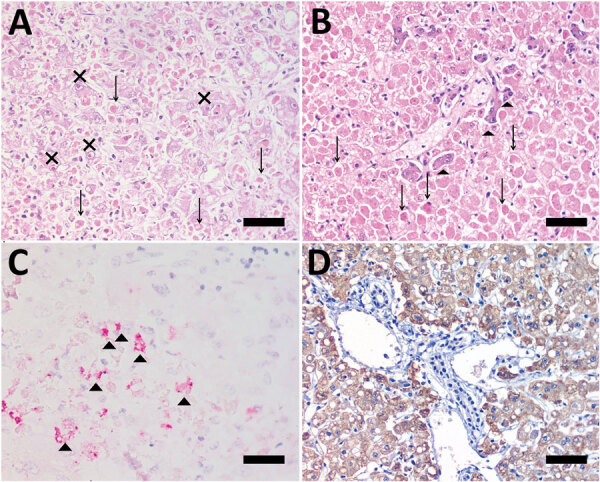
Photomicrographs of liver from human patient (A, C) and *Callicebus* spp. monkey (B, D) with full spectrum of yellow fever (YF)–associated lesions, Brazil. Midzonal necrosis with multiple Councilman-Rocha Lima bodies (arrows), mild steatosis, and hepatocytes with eosinophilic nucleoli (Torres body [Xs]) (hematoxylin and eosin stained). B) Necrosis/apoptosis (diffuse and panlobular), associated with multiple Councilman-Rocha Lima bodies (arrows) and a few remaining viable hepatocytes (arrowheads) in periportal area (hematoxylin and eosin stained). C) Positive, multifocal immunolabeling for YF antigen (arrowheads) (anti-YF, Warp red, counterstained with hematoxylin). D) Intense and diffuse immunolabeling for YF antigen (anti-YF, 3,3'-diaminobenzidine counterstaining with hematoxylin). Scale bars indicate 50 µm.

Microscopic evaluation of IHC-positive cases identified 432 (92.3%) of 468 cases that had a full spectrum of YF hepatic lesions (Figure 1). Three had other histologic patterns: 2 (0.4%) had apoptotic hepatocytes, and 1 (0.2%) had mild degenerative and reaction findings. These 3 cases were *Alouatta* spp. (Appendix Figure 2). Among all positive cases, 33 (7.1%) had autolysis that impaired histologic classification; these cases were excluded from further analysis.

Of all cases evaluated, 1,193 (68.1%) of 1,752 had qRT-PCR results, and 277 (23.2%) of those had detectable YFV: 243 (64.5%) of 374 *Alouatta* spp., 5 (31.2%) of 16 *Callicebus* spp., 5 (7.5%) of 67 *Sapajus* spp., and 24 (3.3%) of 736 *Callithrix* spp. Some cases had inadequate IHC results because of autolysis/decomposition: 2 *Callithrix* spp. and 12 *Alouatta* spp. We found that 24 (8.7%) of 277 (8.7%) qRT-PCR–positive cases had negative results by IHC: 15/24 *Callithrix* spp. (62.5%), 8/243 *Alouatta* spp. (3.3%) and 1/5 *Callicebus* spp. (20%). These cases were called discordant cases, and the remaining cases were called concordant cases.

The discordant group represented, excluding autolyzed tissues, 15 (68.2%) of 22 positive *Callithrix* spp. by qRT-PCR, and 8 (3.5%) of 231 positive *Alouatta* spp. This group included 15 cases with mild degenerative and reactional findings, 4 with steatosis, 1 with non–YF-associated hepatitis, 2 with apoptotic hepatocytes, 1 with multinucleation of hepatocytes, and 1 with no lesions. One case in a *Sapajus* spp. that had a positive IHC result and a negative qRT-PCR result was not considered for statistical analysis because of intense autolysis that impaired morphologic analysis.

**Figure 2:**
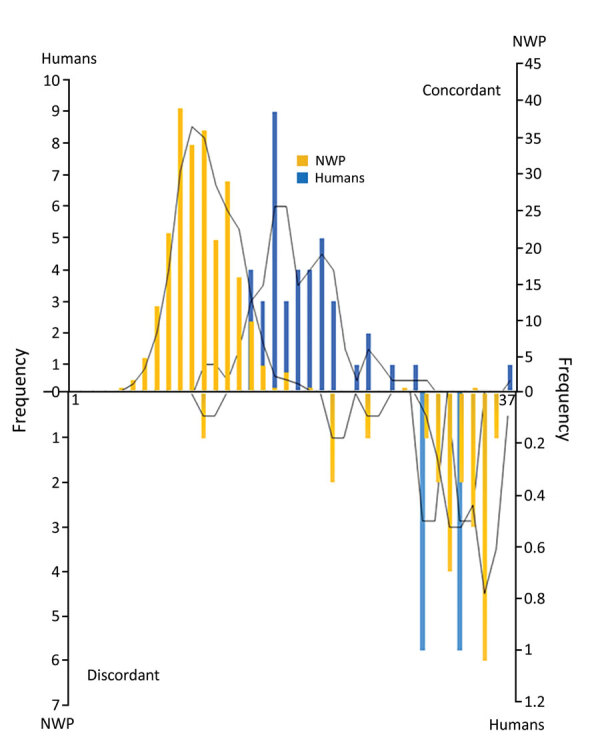
Distribution of Cq values for yellow fever in humans and NWP for concordant and discordant groups, Brazil. Discordant cases had higher Cq values, indicated on right side of the lower histogram, and concordant cases had lower Cq values (higher in humans than in NWP) in the upper histogram. Concordance was determined by using immunohistochemical analysis. The y-axes indicate number of persons and the x-axes indicate Cq value. Cq, quantification cycle; NWP, New World primate.

Regarding viral load, *Callithrix* spp. had a higher Cq (median 34, range 9–37) than other genera (p<0.001). There was no significant difference in Cq for *Alouatta*, *Callicebus*, and *Sapajus* spp. The discordant group had a higher Cq (median 34, range 12–37) than the concordant group (median 12, range 5–35) (p<0.001). When we compared viral load in the concordant group, we observed no difference for NWP genera. We compared Cq values for the concordant and discordant groups (Figure 2) and the distribution of Cq values for *Alouatta* and *Callithrix* spp. and humans (Appendix Figures 3–5).

We calculated YF-associated proportional mortality rates on the basis of cases with liver antigen detected by IHC divided by all cases and YF proportional infections as number of cases with detectable YF virus by qRT-PCR divided by all cases. *Callithrix* sp. was the only genus with difference between proportional YF-related mortality rates and infection (p = 0.01), and proportional YF-related mortality rates and infection were different for NWP genera (p<0.01) (Table 1).

### Humans

We received 93 specimens from human case-patients who had suspected YF, 68 (73.1%) men and 25 (26.9%) women. Of these, 48 (51.6%) had detectable YFV by qRT-PCR, 40 (83.3%) men and 8 (16.7%) women. Of these 48 patients, 46 (95.8%) were IHC positive (concordant) and 2 (4.7%) were IHC negative (discordant). The mean ± SD age for positive case-patients by qRT-PCR (48.8 ± 14.9 years) was higher than for negative case-patients (41.5 ± 18.8 years) (p = 0.04), and a higher prevalence was found in men (p = 0.03). The mean ± SD time in days between onset of clinical signs and time of death (TOD) was 10.7 ± 4.8 (minimum 2 days, maximum 26 days). There was a direct correlation between TOD and Cq (ρ = 0.37; p = 0.007), indicating an inverse correlation between TOD and viral load (Appendix Figure 6).

**Figure 3:**
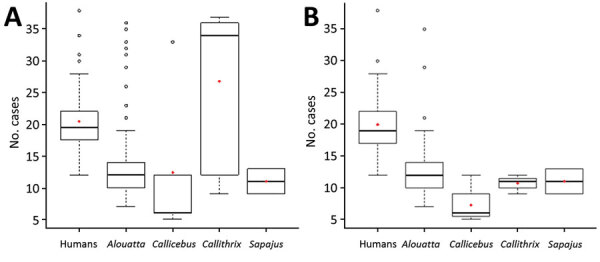
Distribution of Cq values for yellow fever for concordant and discordant cases (A) and cases in different New World primates and in humans (B), Brazil. Box plots indicate Cq values among the groups, indicated by the y-axes, minimum, first quartile, third quartile, and maximum Cq values. Horizontal bars indicate medians. Circles indicate outlier Cq values, and red diamonds indicate mean Cq values. Concordance was determined by using immunohistochemical analysis (lesions and viral antigen in hepatocytes).

All IHC-positive cases showed a full spectrum of YF-associated lesions; 2 discordant cases had other histologic patterns (Appendix Table 3, Figure 7). Regarding viral load, humans had a median Cq of 20 (range 12–38). Human patients had significantly higher Cq values than NWPs in general (p<0.001), except when compared with *Callithrix* spp. When we compared human concordant and NWP concordant cases, humans had a higher Cq than all genera of NWP. We compiled the distribution of Cq values for different NWP genera and humans (Figure 3) and the main characteristics of positive cases among humans and NWPs (Table 2).

**Table 2:** Summary of main findings of human and NWP cases of yellow fever, Brazil*

Category	Human	NWP genus			
		*Alouatta* spp., n = 445	*Sapajus* spp., n = 13	*Callithrix* spp., n = 25	*Callicebus* spp., n = 9
Sex					
M	40	208	5	9	3
F	8	79	5	5	5
Age					
Humans, y					
0–19	0				
20–30	5				
31–45	13				
46–65	24				
>65	7				
NWP, category					
Cub		18	0	1	0
Young		39	4	0	3
Adult		165	5	11	4
Hepatic histology†					
Full spectrum of YF-associated lesions	45	403	12	9	8
Incomplete spectrum of YF-associated lesions	3	11	0	15	1
IHC and RT-qPCR profile					
Concordant	46	223	5	7	4
Discordant	2	7	0	14	1
Quantification cycle					
<10	0	73	2	3	3
11–20	28	147	3	4	1
21–30	17	5	0	0	0
>30	3	5	0	14	1

*IHC, immunohistochemical analysis; NA, not applicable; NWPs, New Word primates; RT-qPCR, reverse transcription quantitative PCR. †Cases with marked autolysis were excluded.

### Comparison of Methods

Histopathologic examination showed high sensitivity and specificity for most genera of NWPs and for human specimens if one considers the full spectrum of YF-associated lesions as positive results (Table 3). Agreement between qRT-PCR and IHC for detection of YFV was good for NWPs (κ = 0.936, 95% CI 0.911–0.961) and humans (κ = 0.957, 95% CI 0.898–1.000). Analysis of NWPs by genera showed that *Callithrix* spp. had lower agreement (κ = 0.475, 95% CI 0.251–0.698) than other genera. 

**Table 3:** Performance of histopathologic analysis in diagnosing yellow fever in NWPs and humans, Brazil*

Category	Sensitivity	Specificity	PPV	NPV	IHC and qRT-PCR agreement, κ
NWPs					
All genera	94.1 (91.6–96.1)	99.5 (98.8–99.8)	98.6 (97–99.4)	97.6 (96.6–98.3)	0.97 (0.91–0.96)
* Alouatta*	97.3 (95.3–98.7)	98.2 (95.6–99.5)	99 (97–99.4)	95.3 (91.9–97.3)	0.95 (0.92–0.98)
* Callithrix*	37.5 (18.8–59.4)	99.9 (99.3–100.0)	90.0 (54.3–98.6)	98.2 (97.5–98.7)	0.47 (0.25–0.70)
* Sapajus*	100 (73.5–100.0)	100 (94.4–100.0)	100 (94.4–100.0)	100.0 (94.4–100.0)	0.90 (0.70–1.00)
* Callicebus*	88.9 (51.7–99.7)	94.7 (74.0–99.9)	88.9 (53.9–98.2)	94.7 (73.9–99.1)	0.85 (0.56–1.00)
Humans	97.9 (88.7–99.9)	97.8 (88.5–99.9)	97.9 (86.9–99.7)	97.8 (86.6–99.7)	0.96 (0.90–1.00)

*Values are % (95% CI). IHC, immunohistochemical analysis; NPV, negative predictive value; NWPs, New Word primates; PPV, positive predictive value; RT-qPCR, reverse transcription quantitative PCR.

The detection limit for IHC was calculated by using receiver operating characteristic curve analysis and considered the Cq value for liver samples. For NWPs in general, the cutoff value was 21 (sensitivity 99.2%, specificity 95.6%). For *Alouatta* spp., the cutoff value was 21 (sensitivity 99.1%, specificity 87.5%); for *Callithrix* spp., the cutoff value was 12 (specificity and sensitivity 100%) (p<0.0001).

## Discussion

A major epidemic of YF occurred in Brazil during 2016–2018. This epidemic had a dramatic progression in São Paulo state, and virus circulated in areas without vaccination coverage (*15*,*19*). Wildlife surveillance is a pivotal tool for understanding arbovirus dynamics, despite difficulties in obtaining high-quality samples for analysis (*20*). A nonhuman primate (NHP) (rhesus monkey) is a useful model for YF (*21*) because it shows lesions similar to those in humans, although comparative studies among animals and humans, especially in an epidemic context, have not been conducted.

We analyzed a specific set of human samples that had a profile compatible with a sylvatic cycle: predominance of middle-age men, as proposed elsewhere (*19*). During 2012–2014, a YF outbreak occurred in the South Omo Zone in Ethiopia, which also had a higher mortality rate for adult men (*22*). However, other urban arboviral disease outbreaks, such as dengue, show similar proportions in both sexes and a lower median age (*23*).

In NWPs, there was no difference in sex and age group distribution between positive and negative cases for YF, although adult males, especially from the genus *Alouatta*, were the most affected. Moreno et al. (*24*) observed the same profile in an outbreak among NWPs in Argentina, which highlighted the impact of conservation and reduction of population growth and mortality rates once adults have an effect on population reproductive capacity and provide care of young animals.

Most human samples had a traditional histologic pattern (positive results for IHC) consistent with YF (*1**,**13**,**14*). Similarly to NWPs, we found discordant results for human case-patients who had lower viral loads, 1 of them limited by histologic over-fixation. Fixation periods >1 week might reduce immunoreactivity in tissues, even for robust antigens, such Ki-67 (*25*). In case-patients who had low viral loads and probably low amounts of antigen, these factors might have a higher negative effect. A direct correlation between TOD and Cq was observed for humans. This finding could explain the higher Cq values for humans than for NWPs because animals were mostly free living and were found dead without having any previous veterinary assistance. Also, the higher viral load among NWPs might indicate the higher potential of them as a group to act as amplifiers compared with humans.

*Alouatta* spp. accounted for >90% of IHC-positive cases; this genus also had the highest proportional mortality rate attributable to YF and was the most affected animal group. *Alouatta* spp. are reported as being highly sensitive to YFV and the major sentinel in YF epidemics; epizootics have been reported in Venezuela (*12*) and Argentina (*24*), which affected howler monkeys and humans. In Brazil during the outbreak of 2008–2009, thousands of howler monkeys died in the southern region (*26*). There was also a major reduction in populations of these animals. In São Paulo state (*8*), both situations led to expansion of the YF vaccine coverage area.

*Callicebus* spp. is one of the most diverse genera of NWP and widely distributed in South America (*27*). Although these monkeys have been described as being resistant to YF (*26*), there are a few reports of YFV, including a genomic sequence extracted from a virus isolated from a monkey of this genus (*28*). We found no information regarding mortality rates or histologic pattern of lesions in the literature. In our study, *Callicebus* spp. monkeys were sensitive to YFV and developed a full spectrum of liver lesions, similar to those in humans, and a high viral load. These manifestations also occurred for *Sapajus* spp. monkeys.

*Callithrix* spp. overrepresented the analyzed animals, although only 1.1% of them died from YF. *Callithrix* is a genus widely distributed in São Paulo state and contains a vulnerable native species, *C. aurita*, a species introduced from northeastern Brazil; *C. jacchus* and *C. penicillata*, which were reeintroduced from the savannah; and natural hybrids (*29*). Regarding YF, most experimental studies date from the 1930s and 1940s in which YFV was transmitted from inoculated *C. penicillata* monkeys to rhesus monkeys through mosquitos. Although some of the experimented animals had fevers and died, none of them had typical hepatic lesions of YF (*30*).

Knowledge about NWP hosts and their susceptibility to YFV could help clarify the potential of maintenance of virus in interepidemic periods among these different genera and anticipate new spillovers or establishment of enzootic cycles. It is useful to consider behavioral changes in human and NWP populations, such as anthropic invasion in natural areas, frequently in forest areas; intensification of agricultural activities; and presence of some genera of NWPs in urban areas. Santos et al. (*31*) reported that *C. penicillata* monkeys are capable of adapting to urban environments; can keep natural behavior and group sizes; and adapt to food sources normally found in these areas. In our study, we found positive callitrichids in urban or periurban areas, as well as *Alouatta* spp. monkeys in parks within urban areas, such as São Paulo. Therefore, the traditional division into 2 YF cycles (sylvatic and urban) might be insufficient or inaccurate to describe the complexity seen in this recent outbreak, and the coexistence of humans, NWPs, and vectors adapted to both groups in the same environment must be considered.

Valentine et al. discussed sylvatic cycles of arboviruses and highlighted the role of NHPs as hosts for chickungunya and Mayaro fever, although the possibility of other NHP reservoirs for both diseases is debated (*32*). The distinction among amplifiers and reservoirs in arbovirus cycles is complex, especially because of the acute nature of infection; short time for viremia and high mortality rates are characteristics not compatible with the classical reservoir definition (*16*).

Viral persistence in a host must not cause death or serious disease, and in short-term infections, such as YFV, the virus must find a new susceptible host within a host population to keep permanent circulation (*33*). Thus, arthropod vectors are considered the most suitable reservoir for arbovirus, and NHPs are commonly described as amplifiers (*16*). The profile exhibited by callitrichids in our study differed from those of other genera, and similar to our findings in dead animals, living animals might have low viremia with no clinical hepatitis. Consequently, the role of callitrichids as amplifiers must be questioned, and their potential as a reservoir population warrants further research.

São Paulo state has a strict range of callitrichid species: *C. penicillata*, *C. jacchus*, *C. aurita*, and hybrid *Callithrix.* These taxonomic variations might be related to differential patterns. A group of animals with high viral loads (similar to the other studied genera) and hepatic lesions could affect general susceptibility in a population. In a surveillance context, callitrichids have a minor role as sentinels because of their low prevalence, although they could be relevant in urban areas, if one considers how adapted they are to these environments and different they are from the other genera analyzed. Discordant cases represent a diagnostic and medical challenge that has clinical and epidemiologic implications, especially in NWPs, because there is usually no available clinical information, such as clinical signs and other laboratory findings (liver markers, blood count). We did not observe any specific liver lesion in these cases.

In our study, callitrichids represented most samples but showed the lowest positivity rates, which was consistent with results of laboratory methods. Better selection of animals for analysis based on geographic locations and host populations from other genera could guide economic and personnel resources and optimize surveillance.

The histologic pattern for YF, especially Councilman-Rocha Lima bodies, was described more than a century ago (*34*), and its role as a diagnostic tool has been reinforced in other reports (*13*,*14*). In our study, histology was a sensitive tool for use with human and NWP samples; callitrichids were the exception. IHC for YF provided etiologic confirmation and high agreement with qRT-PCR results, even for autolyzed cases (*3*).

IHC and qRT-PCR had similar usefulness in diagnosing YF in an epidemic context. Furthermore, an inexpensive analysis, such as histopathologic analysis, could provide useful information, such as excluding negative cases, increasing laboratory response, and detecting other infectious diseases, some of them zoonoses. Moreover, histopathologic analysis, especially IHC, might be a useful screening examination because of its high sensitivity, low cost, and limited human resource requirements. The cases we report as discordant and positive cases with minimal histological lesions illustrate the usefulness of submission of complete epidemiologic and clinical information and samples from different organs for histologic analysis. In addition, a well-trained pathologist with experience in YF diagnosis in NWPs is needed to ensure a sensitive and specific diagnosis on the basis of histologic criteria.

Our study had some limitations. We could not identify species of NWPs, and differential susceptibility among members of the same genus is still unknown. We might have had bias related to sample representation because collection of carcasses was limited to areas near trails or roads. Also, this study was restricted to São Paulo state landscapes and its NWP diversity. Although the genera analyzed are distributed in other geographic areas, more studies with different neotropical primates are needed to ensure ideal surveillance.

In conclusion, NWPs are not a homogenous group and show differences regarding susceptibility, viral load, and proportional YF-associated mortality rates. Clarification of the differences within NWP genera and between NWPs and humans might help to improve and optimize strategic surveillance by directing laboratory resources to most susceptible genera and applying diagnostic tests in a more rational way.

Appendix Additional information on differential yellow fever susceptibility in new world nonhuman primates, comparison with humans, and implications for surveillance, Brazil.
