# Supplementary material for: Differential Yellow Fever Susceptibility in New World Nonhuman Primates, Comparison with Humans, and Implications for Surveillance
Source: Emerg Infect Dis. 2021 Jan;27(1):47–56. doi: 10.3201/eid2701.191220 (PMC7774563; doi:10.3201/eid2701.191220)
Supplement: Appendix — Additional information on differential yellow fever susceptibility in new world nonhuman primates, comparison with humans, and implications for surveillance, Brazil. [file 19-1220-Techapp-s1.pdf]

Article DOI: https://doi.org/10.3201/eid2701.191220

Differential Yellow Fever Susceptibility in New World Nonhuman Primates, Comparison with Humans, and Implications for Surveillance

Appendix

Appendix Table 1. Histologic categories and description of liver microscopic lesions for yellow fever

Category		Lesions comprised
Full spectrum of yellow fever–associated hepatic lesions		Zonal to massive necrosis/apoptosis of hepatocytes and Councilman-Rocha Lima bodies, sometimes associated with steatosis, hemorrhage, and mild inflammatory infiltrate
Other histologic patterns	Mild degenerative and reactional findings	Hepatocyte ballooning or mild inflammatory infiltrate in portal areas
	Bile duct inflammation	Cholangitis and pericholangitis
	Non-yellow fever–associated hepatitis and necrosis	Inflammatory infiltrate affecting parenchyma associated with hepatocyte injury or ischemic necrosis
	Apoptotic hepatocytes	Scattered apoptotic hepatocytes without inflammation or extensive associated necrosis
	Steatosis	Nonstained cytoplasmic vacuoles in hepatocytes
	Multinucleation of hepatocytes	Hepatocytes with ≥2 nuclei associated with hydropic degeneration
	No lesions	Absence of lesions

Appendix Table 2. Age and sex distribution of New World primates, by genera*

Variable	*Alouatta* spp.	*Callicebus* spp.	*Callithrix* spp.	*Sapajus* spp.	Total
Female	123 (17.4)	9 (32.1)	262 (28.4)	19 (20.0)	413 (23.6)
Adult	83 (11.7)	4 (14.3)	154 (16.7)	10 (10.5)	251 (14.3)
Cub	4 (0.6)	0 (0)	19 (2.1)	0 (0)	23 (1.3)
Young	11 (1.6)	3 (10.7)	36 (3.9)	3 (3.2)	53 (3.0)
Age not identified	25 (3.5)	2 (7.1)	50 (5.4)	4 (4.2)	81 (4.6)
Senile	0 (0)	0 (0)	3 (0.3)	2 (2.1)	5 (0.3)
Male	302 (42.7)	10 (35.7)	285 (30.9)	41 (43.2)	638 (36.4)
Adult	159 (22.5)	6 (21.4)	163 (17.7)	23 (24.2)	351(20.0)
Cub	24 (3.4)	1 (3.6)	22 (2.4)	2 (2.1)	49 (2.8)
Young	55 (7.8)	0 (0)	35 (3.8)	8 (8.4)	98 (5.6)
Age not identified	64 (9.0)	3 (10.7)	63 (6.8)	7 (7.4)	137 (7.8)
Senile	0 (0)	0 (0)	2 (0.2)	1 (1.1)	3 (0.2)
Sex not identified	283 (40.0)	9 (32.1)	374 (40.6)	35 (36.8)	701 (40.0)
Adult	12 (1.7)	0 (0)	15 (1.6)	6 (6.3)	33 (1.9)
Cub	1 (0.1)	0 (0)	5 (0.5)	0 (0)	6 (0.3)
Young	0 (0)	0 (0)	7 (0.8)	2(2.1)	9 (0.5)
Age not identified	270 (38.1)	9 (32.1)	347 (37.7)	27 (28.4)	653 (37.3)
Total	708 (100.0)	28 (100.0)	921 (100.0)	95 (100.0)	1,752 (100.0)

Values are no. (%).

Appendix Table 3. Liver microscopic findings and other clinically relevant information from discordant cases of yellow fever in humans*

Human case-patient	Histologic hepatic findings	Other relevant information
1	Microvesicular and macrovesicular steatosis associated with moderate hemosiderosis and mild hepatocyte ballooning	Cq = 34; splenic hemosiderosis and severe acute necrotizing pancreatitis; lipase level >500 U/L; TOD = 2 d
2	Hepatocyte necrosis associated with ductular reaction	Cq = 31; TOD = 20 d; long-term storage of specimen in formalin reported

*Histologic and immunohistochemical analysis photomicrographs are shown in Appendix Figure 7. Cq, quantification cycle; TOD, time between onset of clinical signs and death.

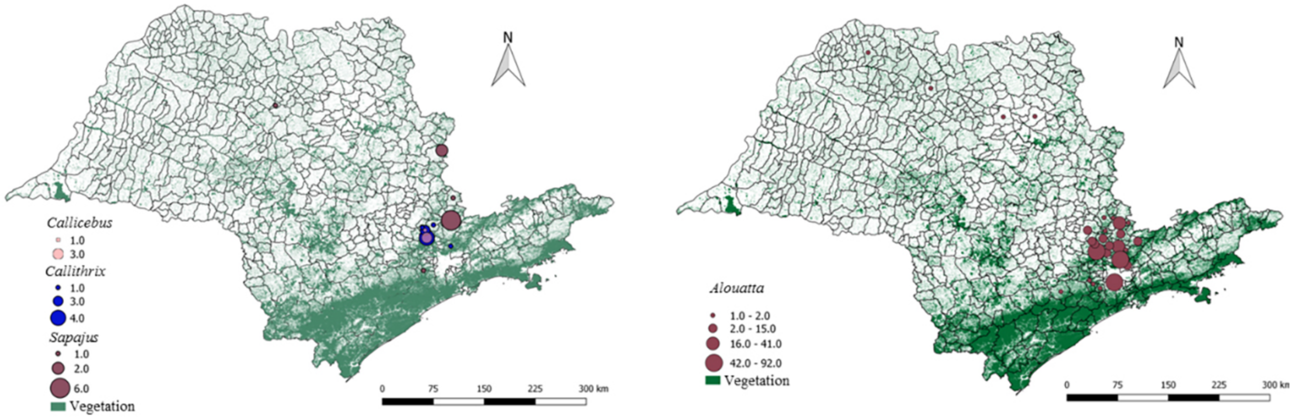

Appendix Figure 1. Spatial distribution of New World primates with yellow fever, by genera, São Paulo, Brazil

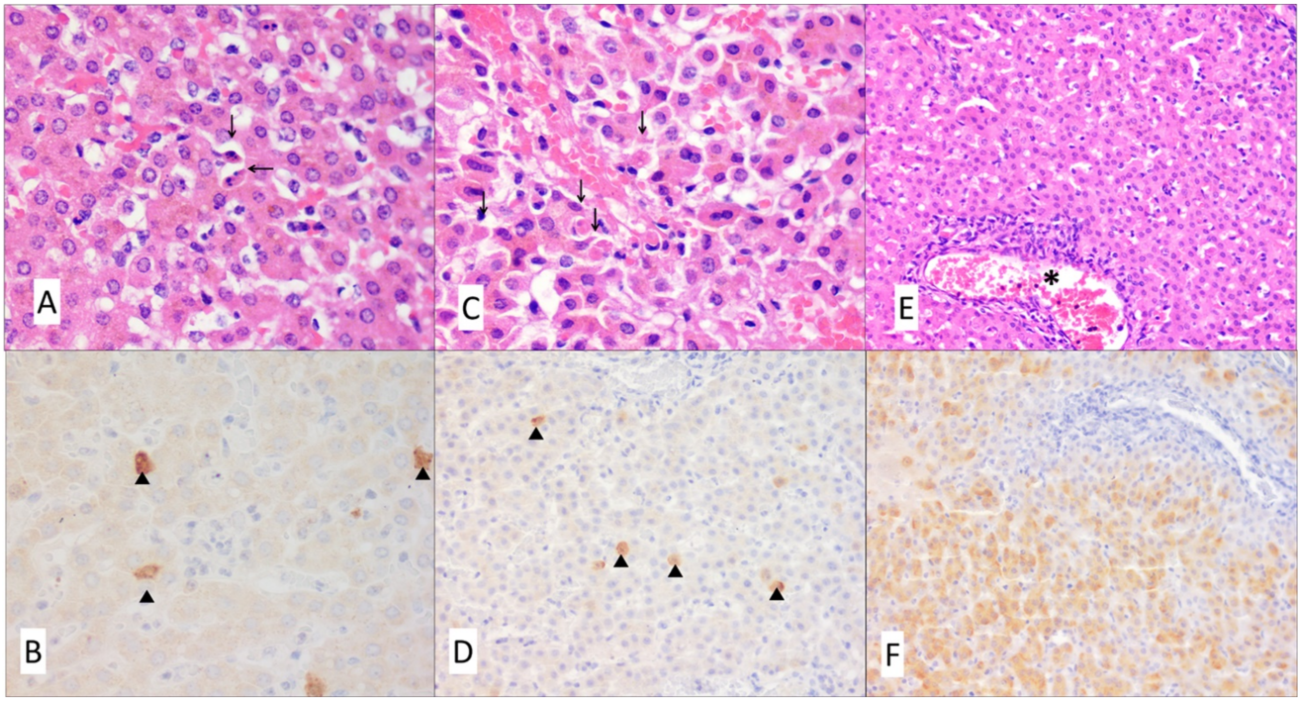

Appendix Figure 2. Photomicrographs of livers from 3 *Alouatta* spp. with nontypical findings of yellow fever. A and C) Rare apoptotic cells (arrows) scattered through hepatic parenchyma (hematoxylin and eosin stained, original magnification x400. B and D) Mild and multifocal immunolabeling for yellow fever antigen (arrowheads)

(immunohistochemical staining with anti-YF, counterstaining with hematoxylin). Original magnification x400 for B and x200 for D. E) Mild reactions showing a lymphohistiocytic infiltrate in portal area (*) (hematoxylin and eosin stained, original magnification x200). F) Intense and panlobular immunolabeling for yellow fever antigen (immunohistochemical staining with anti-YF, counterstaining with hematoxylin, original magnification x400).

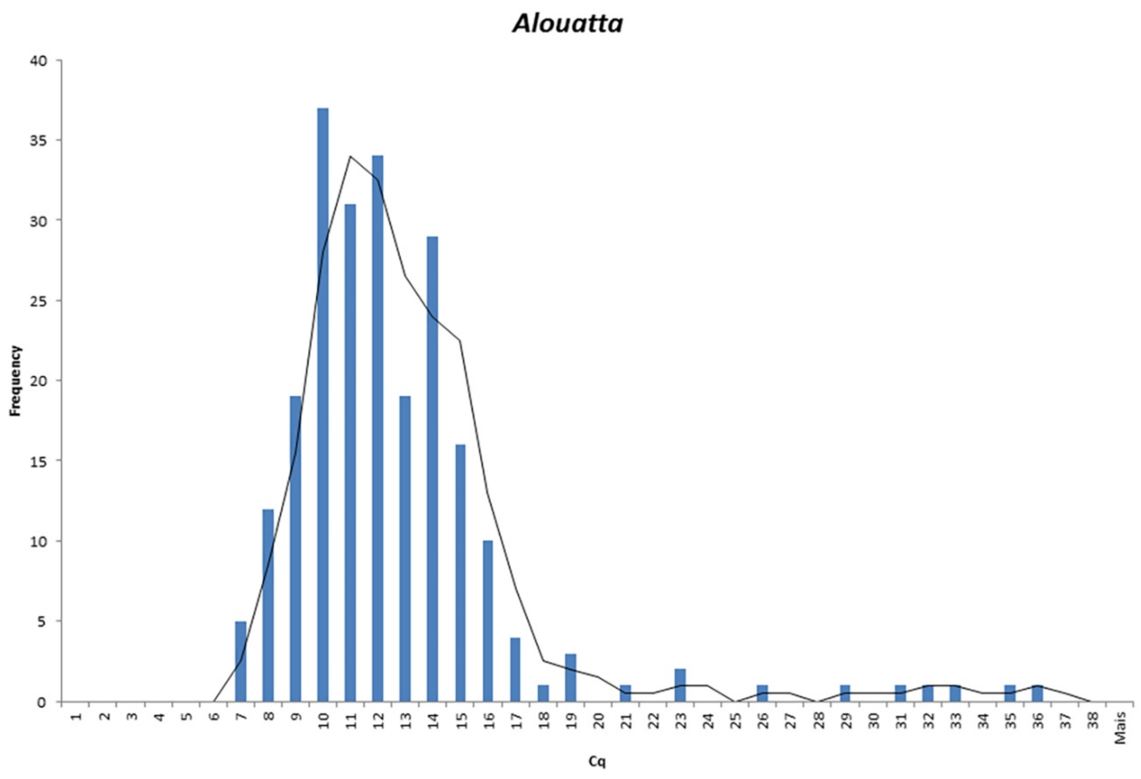

Appendix Figure 3. Histogram showing Cq value distribution for *Alouatta* spp. Cq, quantification cycle.

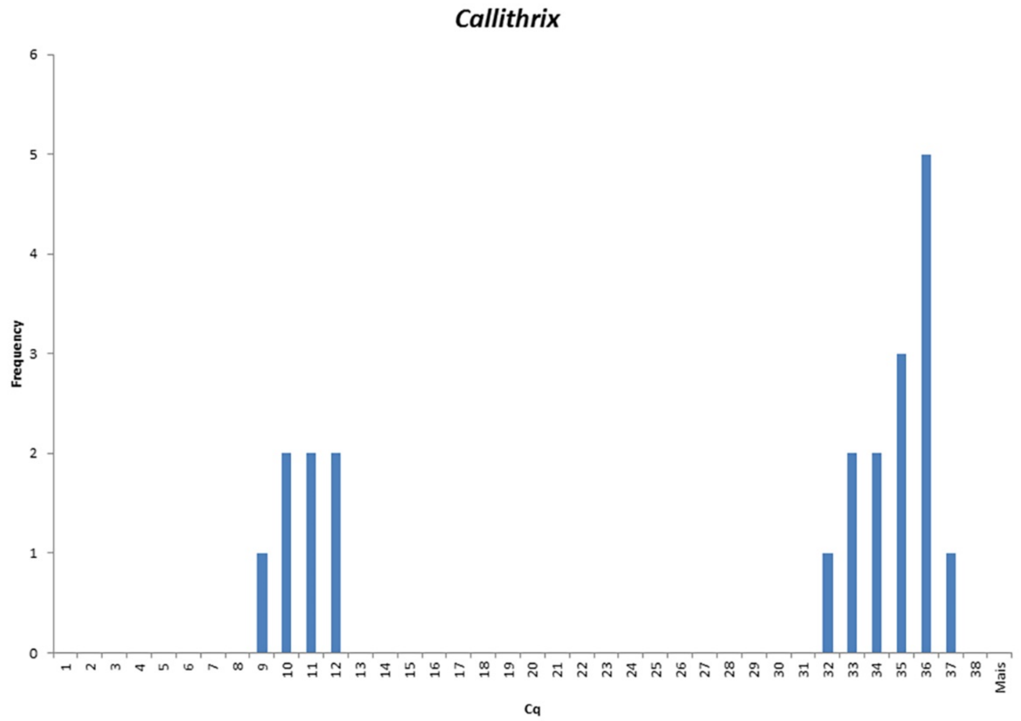

Appendix Figure 4. Histogram showing Cq value distribution for *Callithrix* spp. Callitrichids showed a bimodal distribution, with a group of cases with low Cq values and other group with high Cq values. Cq, quantification cycle.

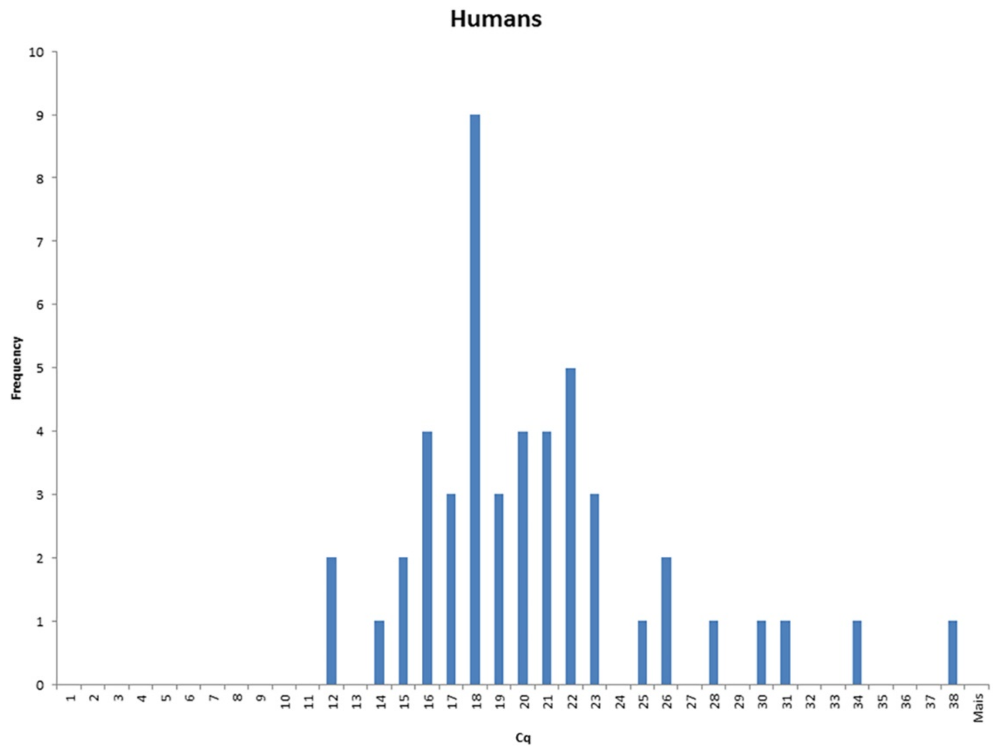

Appendix Figure 5. Histogram showing Cq value distribution for human samples. Cq, quantification cycle.

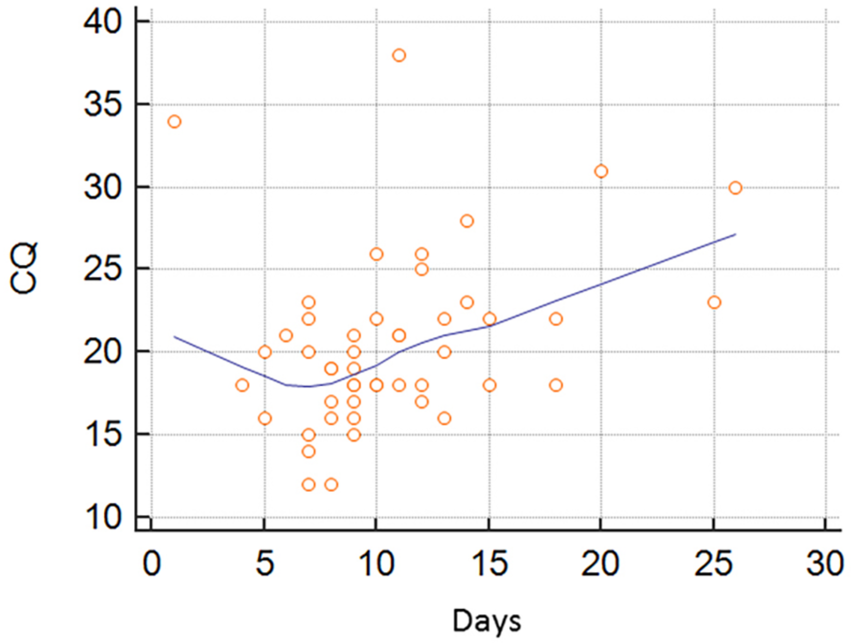

Appendix Figure 6. Correlation (blue line) between Cq value and time in days between onset of clinical signs and death for human case-patients (red circles) who had yellow fever. Early onset of clinical signs has a moderate positive correlation with higher viral load (ρ = 0.37, p = 0.007). Cq, quantification cycle.

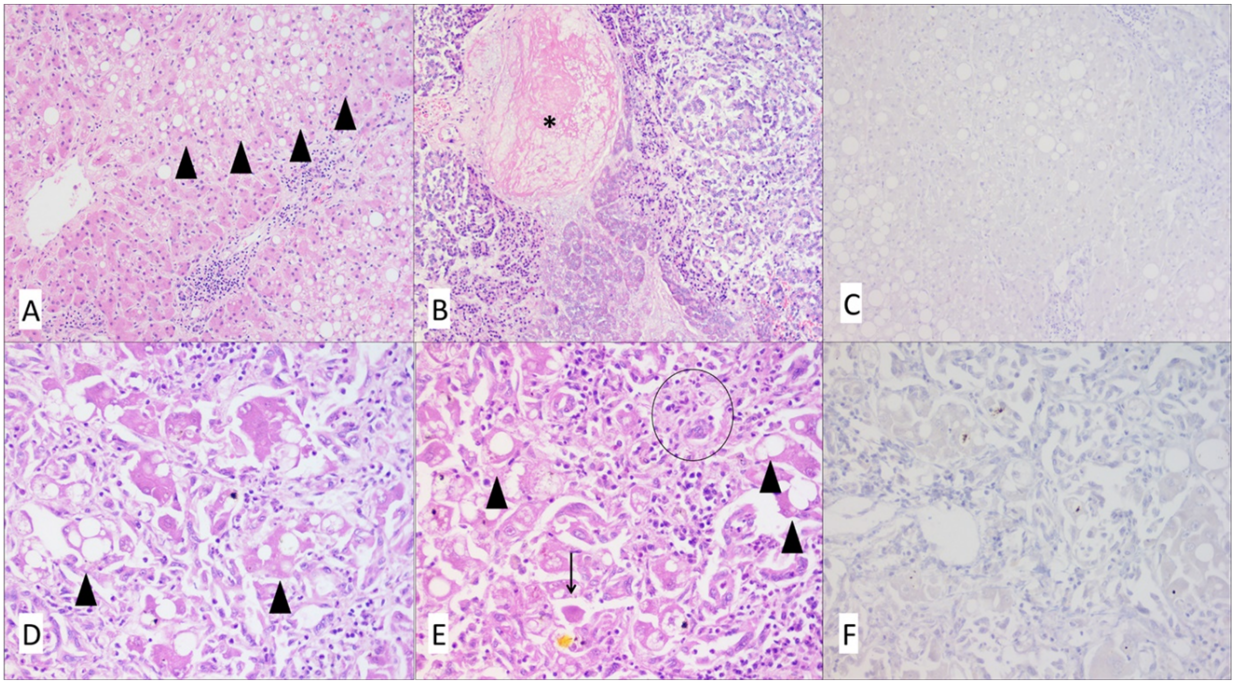

Appendix Figure 7. Photomicrographs of organs from 2 human patients with yellow fever and negative IHC and positive RT-qPCR results (discordant). A, B, and C) Patient 1 (Appendix Table 2). A) Liver showing midzonal macrovesicular and microvesicular steatosis (arrowheads) associated with mild portal inflammatory infiltrate (hematoxylin and eosin stained, original magnification x100). B). Pancreas showing necrosis with thrombi (*) (hematoxylin and eosin stained, original magnification x100). C) Liver showing negative immunolabeling for yellow

fever antigen (immunohistochemical staining with anti-YF, counterstaining with hematoxylin, original magnification x100). D, E, and F) patient 2 (Appendix Table 2). D) Liver showing necrotic hepatitis associated with moderate lymphohistiocytic infiltrate and mild steatosis (arrowheads) (hematoxylin and eosin stained, original magnifications x200). E) Liver showing necrotic hepatitis with evidence of ductular reaction and mild steatosis (arrowheads). F) Liver showing negative immunolabeling for YF antigen (immunohistochemical staining with anti-YF, counterstaining with hematoxylin).
